# Antagonist properties of monoclonal antibodies to human CD28: role of valency and heavy-chain constant domain

**DOI:** 10.1186/1479-5876-10-S3-P17

**Published:** 2012-11-28

**Authors:** Caroline Mary, Flora Coulon, Nicolas Poirier, Nahzli Dilek, Bernard Martinet, Gilles Blancho, Bernard Vanhove

**Affiliations:** 1 Institut National de la Santé et de la Recherche Médicale Unité Mixte de Recherche-Santé 1064, Nantes, France; 2 Effimune SAS, Nantes, France; 3 Institut de Transplantation Urologie Néphrologie, Centre Hospitalier Universitaire de Nantes, Université de Nantes, Nantes, France

## Background

Antagonist antibodies targeting CD28 have been proposed as an alternative to the use of CD80/86 antagonists to modulate T cell responses in autoimmunity and transplantation. Advantages would be the blockade of CD28-mediated co-stimulatory signals without impeding co-inhibitory signals depending on CD80 interactions with CTLA-4 and PD-L1 that are important for the control of immune responses and for the function of regulatory T cells. Anti-CD28 antibodies are candidate antagonists only if they prevent access to the CD80/86 ligands without simultaneously stimulating CD28 itself, a process that is believed to depend on receptor multimerization.

## Methods and results

In this study, we used different formats of a potentially antagonist anti-human CD28 antibody and evaluated the impact on T cell activation of valency and of the presence of a fragment cristallisable (Fc) domain, two components that might impact receptor multimerization either directly or in the presence of accessory cells expressing Fc receptors. Among monovalent (Fab’, scFv), divalent (Fab’2), monovalent-Fc (Fv-Fc) and divalent-Fc (IgG) formats, only the monovalent formats showed consistent absence of induced CD28 multimerization and of associated activation of Phospho Inositol-3 Kinase as well as clear antagonist properties in T cell stimulation assays. In contrast divalent antibodies showed agonist properties resulting in cell proliferation and cytokine release, in a Fc-independent manner. Conjugation of monovalent antibodies with polyethylene glycol, with a molecule of alpha-1-antitrypsin or with an Fc domain significantly extended their in vivo half-life without modifying antagonist properties.

## Conclusion

These data indicate that monovalency is mandatory for maintaining antagonistic activity of anti-CD28 monoclonal antibodies.

